# Molecular cloning of the gene promoter encoding the human Ca_V_γ_2_/Stargazin divergent transcript (*CACNG2-DT*): characterization and regulation by the cAMP-PKA/CREB signaling pathway

**DOI:** 10.3389/fphys.2023.1286808

**Published:** 2023-11-16

**Authors:** David Muñoz-Herrera, Aida Calderón-Rivera, Natanael Zarco, Alejandra Corzo-Lopez, Margarita Leyva-Leyva, Eduardo Monjaraz, Alejandro Sandoval, Norma Oviedo, Ricardo González-Ramírez, Ricardo Felix

**Affiliations:** ^1^ Department of Cell Biology, Centre for Research and Advanced Studies (Cinvestav), Mexico City, Mexico; ^2^ Department of Molecular Biology and Histocompatibility, “Dr. Manuel Gea González” General Hospital, Mexico City, Mexico; ^3^ Institute of Physiology, Meritorious Autonomous University of Puebla (BUAP), Puebla, Mexico; ^4^ School of Medicine FES Iztacala, National Autonomous University of Mexico, Mexico City, Mexico; ^5^ Unidad de Investigación Médica en Inmunología e Infectología, Centro Médico Nacional La Raza, Instituto Mexicano del Seguro Social (IMSS), Mexico City, Mexico

**Keywords:** Ca_V_γ_2_, Cav channels, *CACNG2*, AMPA receptor, CREB, stargazin, TARPγ_2_

## Abstract

Ca_V_γ_2_ (Stargazin or TARPγ_2_) is a protein expressed in various types of neurons whose function was initially associated with a decrease in the functional expression of voltage-gated presynaptic Ca^2+^ channels (Ca_V_) and which is now known to promote the trafficking of the postsynaptic α-amino-3-hydroxy-5-methyl-4-isoxazole propionic acid receptors (AMPAR) towards the cell membrane. Alterations in Ca_V_γ_2_ expression has been associated with several neurological disorders, such as absence epilepsy. However, its regulation at the transcriptional level has not been intensively addressed. It has been reported that the promoter of the *Cacng2* gene, encoding the rat Ca_V_γ_2_, is bidirectional and regulates the transcription of a long non-coding RNA (lncRNA) in the antisense direction. Here, we investigate the proximal promoter region of the human *CACNG2* gene in the antisense direction and show that this region includes two functional cAMP response elements that regulate the expression of a lncRNA called *CACNG2-DT*. The activity of these sites is significantly enhanced by forskolin, an adenylate cyclase activator, and inhibited by H89, a protein kinase A (PKA) antagonist. Therefore, this regulatory mechanism implies the activation of G protein-coupled receptors and downstream phosphorylation. Interestingly, we also found that the expression of *CACNG2-DT* may increase the levels of the Ca_V_γ_2_ subunit. Together, these data provide novel information on the organization of the human *CACNG2-DT* gene promoter, describe modulatory domains and mechanisms that can mediate various regulatory inputs, and provide initial information on the molecular mechanisms that regulate the functional expression of the Ca_V_γ_2_ protein.

## Introduction

Glutamate AMPA receptors (AMPAR) mediate much of the excitatory neurotransmission in the nervous system. The functional expression of AMPAR in the synapse is crucial in several forms of synaptic plasticity, including long-term potentiation and depression (Diering and Huganir, 2018; Kamalova and Nakagawa, 2021; Matthews et al., 2021). Therefore, regulating the membrane expression of these proteins allows them to fulfill their highly specialized neuronal functions.

From a molecular point of view, AMPA receptors are homo- or heteromeric complexes of ion-conducting subunits known as GluA1-4 that consist of four domains. The extracellular N-terminal domain, whose function is the assembly of the subunits and the location of receptors at the synapsis; the ligand binding domain, which undergoes conformational changes upon binding to glutamate that result in channel activation; the transmembrane domain, which forms the membrane’s ion channel and, the cytoplasmic C-terminal domain that regulates anchoring, trafficking, and cell signaling. Furthermore, native AMPARs are associated with auxiliary subunits such as the transmembrane AMPAR regulatory proteins (TARPs), which add functional diversity to the channels and contribute to determining their cellular localization (Payne, 2008; Kamalova and Nakagawa, 2021).

In this context, Ca_V_γ_2_, also known as Stargazin (Stg) or TARPγ_2_, is a multifunctional neuronal protein whose heterologous expression was initially shown to inhibit the functional expression of high voltage-activated calcium (Ca_V_) channels (Letts et al., 1998; Kang et al., 2001; Sandoval et al., 2007). These channels couple cell membrane depolarization to Ca^2+^ influx, and in the brain play a central role in the entry of Ca^2+^ required in the nerve terminals to release neurotransmitters (Lacinova, 2005; Felix, 2006; Gandini and Felix, 2015; Zamponi et al., 2015). However, shortly after this idea was challenged when it was found that Ca_V_γ_2_/Stg was capable of regulating the targeting of the AMPAR to the cell membrane (Payne, 2008; Greger et al., 2017; Kamalova and Nakagawa, 2021).

Human Ca_V_γ_2_/Stg is an integral membrane protein of 323 amino acids (∼36 kDa) conformed by four transmembrane segments with amino and carboxyl termini located intracellularly. Notably, this protein is absent in the Stargazer mutant mouse, an animal model of epilepsy with absence seizures and cerebellar ataxia (Letts et al., 1998; Kang et al., 2001).

Molecular studies have shown that the human *CACNG2* gene is located on the antisense strand of chromosome 22, is about 140 kb in size, comprises four exons, and gives rise to an approximately 2.4 kb messenger (Letts et al., 1998). However, little is known regarding the molecular mechanisms that control its expression. The rat *Cacng2* promoter was recently cloned, and its fundamental features include multiple RE-1 silencing transcription factor (REST) domains and a Ca^2+^ regulatory element-binding factor (CaRE) element (Corney et al., 2019). It also regulates the transcription of Ca_V_γ_2_/Stg in the antisense direction of the genome, while in the sense direction controls the transcription of a lncRNA (Corney et al., 2019).

Here, we provide novel information on the bidirectional character of the human *CACNG2* promoter and its organization by describing two novel functional CRE modulatory domains, the presence of a transcription start site (TSS), and a TATA box that may regulate the transcription of a lncRNA called *CACNG2-DT*. Given the presence of functional CRE sites, it was reasonable to propose a possible modulation through the activation of a G protein-coupled receptor. It is well known that when this type of receptors is activated by its ligand, a nucleotide exchange occurs in the Gα subunit, and a conformational change is induced in the Gαβγ complex that results in its dissociation into two active signaling molecules, the Gα-GTP subunit and the Gβγ dimer (Corney et al., 2019). The Gα-GTP subunit, in turn, activates a wide range of signaling pathways that include the participation of adenylate cyclase (AC) and several protein kinases that phosphorylate a wide variety of effectors. Here we show that the activity of the CRE sites in the proximal promoter encoding *CACNG2-DT* is enhanced by forskolin, an activator of AC, acting through p-CREB, the phosphorylated form of the cAMP response element-binding (CREB). In sharp contrast, in the presence of H89, a PKA inhibitor, the transcriptional activity of the promoter is inhibited significantly as a consequence of a reduction in the levels of phosphorylated p-CREB.

Finally, it is also known that lncRNAs are >200 bp RNA molecules that are not translated (Guttman and Rinn, 2012; Hombach and Kretz, 2016; Bridges et al., 2021; Felix et al., 2022) and may have different regulatory functions in the transcription of various genes and the translation of several proteins, including ion channel subunits (Felix et al., 2022). Interestingly, here we show that *CACNG2-DT* may increase the expression of Ca_V_γ_2_/Stg, providing relevant information into the molecular mechanisms that regulate the expression of this intriguing synaptic protein.

## Materials and methods

### In silico analysis

The sequence of the *CACNG2-DT* gene promoter was determined by aligning a 1.3 kb 5′UTR region of the *CACNG2* gene and the *CACNG2-DT* sequence deposited at NCBI (Gene ID: 105373021) using the Align Sequences Nucleotide BLAST software (https://blast.ncbi.nlm.nih.gov/Blast.cgi). The location of putative TSS was achieved using the Neural Network Promoter Prediction tool available on the Berkeley Drosophila Genome Project website (http://www.fruitfly.org/seq_tools/promoter.html). The presence of the TATA box and the possible binding sites for transcription factors and other relevant regulatory elements were determined using the Genomatix MatInspector tool (https://www.genomatix.de/). Finally, sequence analysis was performed with the Jaspar2022 tool (https://jaspar.genereg.net/) to identify possible CREB transcription factor binding sites.

### Deletional analysis and removal of regulatory elements

Different *CACNG2-DT* promoter constructs (A, B, and C) and combinations of these (AB and BC) were obtained by PCR and cloned into the pGL3-Basic vector (Promega) (Supplementary Table S1). The contribution of the putative TSS and CREB-binding sites to the promoter activity was assessed by mutagenesis using the QuikChange II XL Site-Directed Mutagenesis Kit (Agilent Technologies) and specific oligonucleotides (Supplementary Table S2). The cloning of the constructs in the vectors, the elimination of the regulatory elements of the TSS site were corroborated by the alignment of the sequences with the sequence of the *CACNG2-DT* promoter in the NCBI database using the AlignX software from the Vector NTI Advance 11.5.0 bioinformatics package (Invitrogen). All constructs used in this study were automatically sequenced.

### Cell culture and transfection

Transfections were performed in the mouse neuroblastoma-derived cell line N1E-115 (ATCC). Cells were grown in DMEM medium, supplemented with 10% FBS, 100 U/mL penicillin, 100 μg/mL streptomycin, and 2 mM L-glutamine. Where indicated, experiments were performed in SH-SY5Y human neuroblastoma cells (ATCC) maintained in DMEM medium supplemented, as mentioned above. Both cell lines were kept in culture at 37°C in an atmosphere with 5% CO_2_. Cells were kept in culture to 80% confluence and transfected 48 h before being used, using the Lipofectamine 2000 Reagent according to the manufacturer’s instructions (Invitrogen) using 35 mm diameter culture dishes with 2 μg of each plasmid DNA encoding Ca_V_γ_2_/Stg or CREB.

In the case of experiments in which the endogenous adenosine receptor was activated, N1E-115 cells were transfected with the promoter region of the *CACNG2-DT* gene cloned into the pGL3-Basic vector (2 µg) and cotransfected with the plasmid pRL-CMV as control (0.5 µg), using Lipofectamine and Reagent Plus. Five h later, the medium was replaced with medium supplemented with 250 µM adenosine (Merk, Cat. #A4036). Forty-eight h after transfection, the promoter activity assays were performed using the Dual-Glo Luciferase Assay System (Promega) according to the suppliers’ instructions. Luminescence was measured using a luminometer (Modulus-Turner Biosystems). The firefly/*Renilla* luciferase activity ratio was used to normalize the data (see *Statistical analysis*).

### CREB activation and silencing

The activated CREB (p-CREB) levels were estimated by incubating the cells with 3 mM 8-Br-cAMP (Sigma-Aldrich, Cat. #B6272). Also, cells were transfected with a construct containing the human CREB1 coding sequence (pcDNA3-CREB1) to increase the amount of CREB. Likewise, the activation of the adelynate cylase was achived by incubating the cells with 10 μM Forskolin (Sigma-Aldrich). On the other hand, the decrease in p-CREB levels was also achieved by incubating the cells with 10 µM H89 (Sigma-Aldrich), a Protein Kinase A (PKA) inhibitor, or by silencing the transcription factor using small interference RNAs (siRNAs) (Supplementary Table S3).

### Luciferase assays

Constructs cloned into the pGL3-Basic vector were transfected in N1E-115 cells together with the pRSV-βgal plasmid (Promega). The pGL3-Basic vector contains the coding sequence for luciferase from *Photinus pyralis* but lacks a promoter. The pRSV-βgal plasmid contains the coding sequence for β-galactosidase, which was used to normalize the results based on transfection efficiency. The Luciferase Assay System kit (Promega) was used for the assay. The *CACNG2-DT* promoter was also cloned into the pGL4Luc-RLuc vector (Addgene), which has the genes encoding the *Photinus pyralis* and *Renilla reniformis* luciferases separated by ∼100 bp, the region where the sequence of *CACNG2-DT* promoter was cloned. Hence, the synthesis of *Photinus* luciferase depended on the ability of the promoter to initiate transcription, while the expression of *Renilla* luciferase was associated with the antisense promoter transcriptional activity. The Dual-Glo Luciferase Assay System kit (Promega) was used for these assays.

### Protein extraction and western blot

Cells were washed with PBS and incubated in lysis buffer (SDLB) containing 250 mM Tris-HCl [pH 8], 750 mM NaCl, 5% Triton x-100, 1 mM phenylmethylsulfonyl fluoride (PMSF) and the protease inhibitor cocktail Complete 1X (Roche diagnostics). Cells were then incubated at 4°C for 30 min, centrifuged, and the proteins in the supernatant quantified using the Protein Assay Dye Reagent Concentrate (Bio-Rad). Protein expression levels were determined by Western blot. To this end, 50 μg of proteins in Laemmli buffer were heated at 95°C for 5 min. The samples were loaded on a 10% SDS-PAGE gel, and the proteins were separated by electrophoresis using electrophoresis buffer (0.025 M Tris-Base; 0.192 M Glycine; 0.1% SDS). Next, the proteins were semi-dry transferred to nitrocellulose membranes. Membranes were washed with TBST buffer (TBS-T; 10 mM Tris–HCl pH [7.6], 15 mM NaCl, 0.05% Tween 20), and non-specific binding was blocked using TBST with milk (5%). Membranes were then incubated overnight with primary antibodies anti-CREB (1:1000; Cell Signaling), anti-p-CREB (1:1000; Cell Signaling), anti-Ca_V_γ_2_/Stg (1:1000; Santa Cruz) or anti-β-actin (1:10,000, GeneTex) diluted in TBST. Next, membranes were washed with TBST and incubated with anti-mouse (1:5000; Jackson Immunoresearch) or anti-rabbit (1:5000; Jackson Immunoresearch) secondary antibodies coupled to horseradish peroxidase for 1 h. Finally, membranes were developed using the Immobilon Western reagent kit (Millipore). The chemiluminescence signal was detected with the Odyssey Fc kit (LI-COR).

### Rapid amplification of cDNA ends (RACE)

This technique was used to determine the TSS in the *CACNG2-DT* promoter, using the 5′RACE System for Rapid Amplification of cDNA Ends kit (Invitrogen). Total RNA samples were isolated from SH-SY5Y cells and human cerebellum (obtained from Cinvestav’s Brain Bank; https://cgse.cinvestav.mx/Banco-Nacional-de-Cerebros) with TRIzol Reagent (Ambion). The use of this tissue sample was a proved by the Biosafety Committee of the Center for Research and Advanced Studies of the National Polytechnic Institute (protocol No. 2023-1-CB). Oligonucleotides targeting the *CACNG2-DT* (Supplementary Table S4) were used, and the amplicon was cloned into the pCRTM2.1-TOPO vector.

### Statistical analysis

Data were analyzed using GraphPad Prism 8.0.2 software (GraphPad Software). Results are presented as mean ± SEM. Student’s t-test was used to compare two groups, while for the data analysis from several groups, ANOVA followed by Tukey’s *post hoc* test was used. Differences were considered statistically significant with *p* values < 0.05.

Normalization of the data in the luciferase assays was performed as follows, considering that two different systems were used. In the first case, the cells were transfected with a construction consisting of a vector that uses the *Photinus pyralis* firefly luciferase as a reporter gene, where the promoter of the *CACNG2-DT* gene was cloned. In parallel, the cells were cotransfected with an empty vector (without the promoter of interest) that only expresses the luciferase from sea pansy *Renilla reniformis* as a reporter gene, which was used to normalize the data and thus eliminate the transfection efficiency as a variable. The second system also used the very same construct that contains the *Photinus* luciferase as a reporter gene and the promoter of *CACNG2-DT*; however, in this case, the cells were cotransfected with an empty vector that expresses the enzyme β-galactosidase, which, as in the previous case, was used to normalize the data with the same purpose of eliminating transfection efficiency as a variable (see Supplementary Table S5). Note that after the analysis, the scale of the graphs differs by several orders of magnitude due to the nature of the data normalization. The results of these experiments are shown as bar graphs that further display the dispersion of the experimental data.

## Results

**FIGURE 1:**
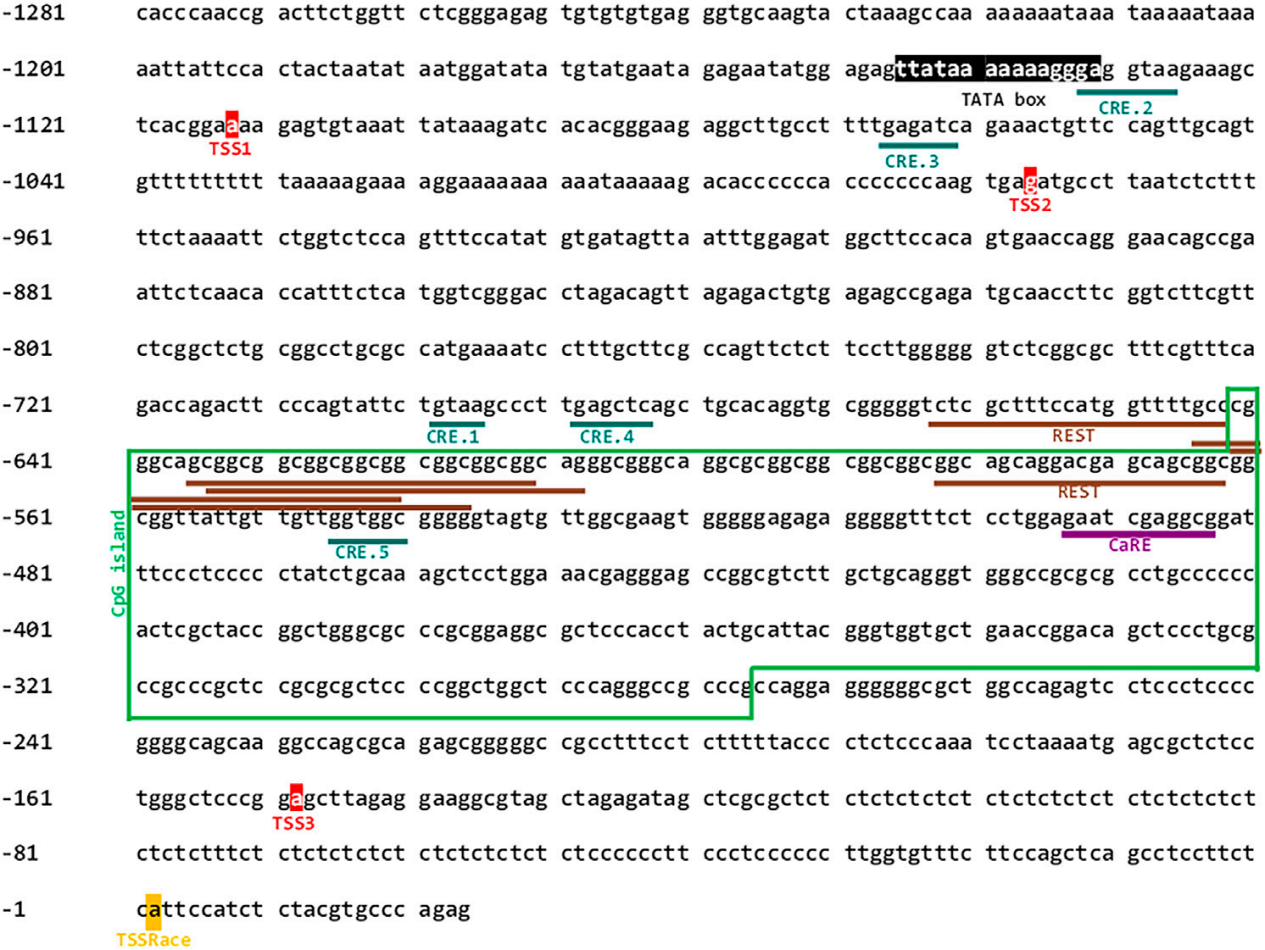
Nucleotide sequence of the *CACNG2-DT* gene promoter. The CREB transcription factor binding sites, the multiple repressive domains of REST and CaRE transcription factor binding site are underlined. Putative signals for the basal transcription machinery (TATA box, TSSs), are also depicted. The green box frames the CpG island.

Previously, it was reported that the rat *Cacng2* gene promoter has bidirectional activity and that differentiation of mouse hippocampal HT22 cells with cAMP increases the mRNA levels of both *Cacng2* and the divergent transcript, which is a lncRNA (Corney et al., 2019). Therefore, we sought to study in detail the human promoter of the human divergent transcript (*CACNG2-DT*). To this end, we initially performed an *in silico* analysis to identify elements that could be regulating its activity. Next, the DNA of interest located between nucleotides 36702595 and 36703898 of the reference sequence in the NCBI database (RS_2023_03) was cloned into the pGL3-Basic vector. The cloned region is 1304 bp in size, 1158 corresponding to the 5′UTR region of the *CACNG2* gene; an intergenic region of 123 bp, and a small sequence of 23 bp of exon 1. Interestingly, a TATA box located at position -1147 and -1133 was also found, as well as three nucleotides located at positions -1114, -978, and -150 that might function as TSS. Last, a CpG island, a common feature of bidirectional promoters, between nucleotides -643 and -278 was also identified (Figure 1). The identification number of the *CACNG2-DT* gene in the NCBI database is 10537302.

**FIGURE 2:**
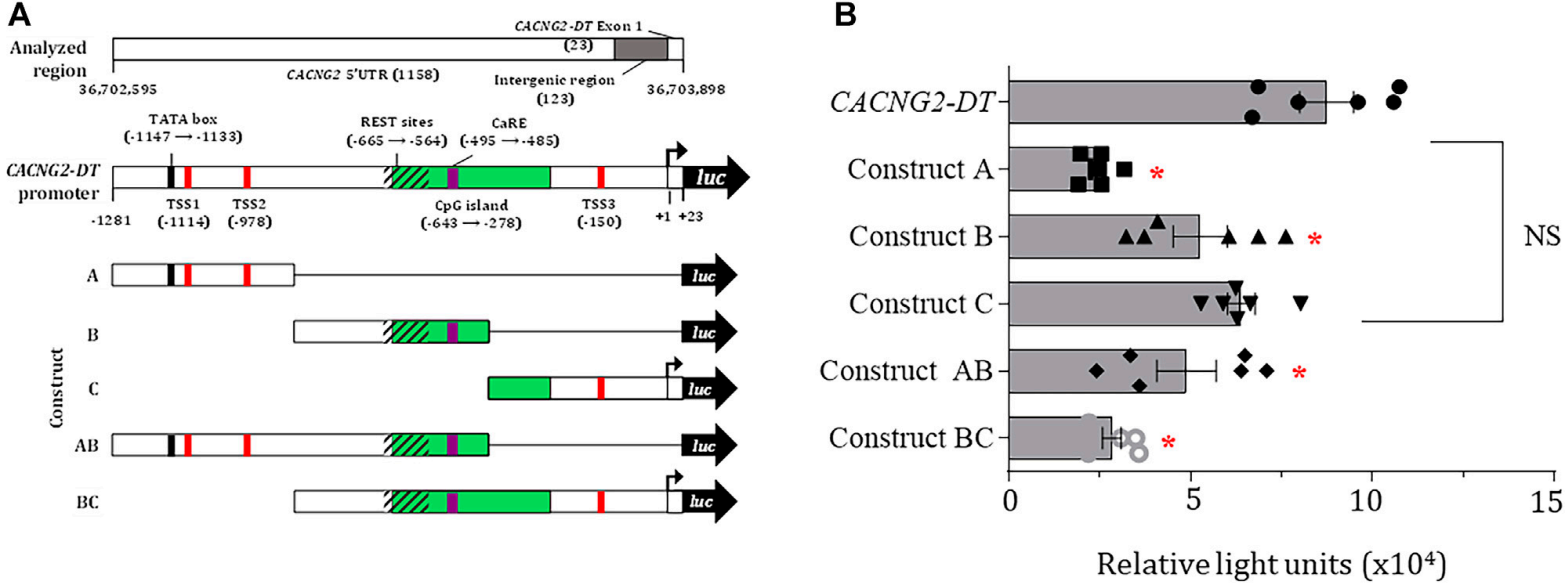
Deletional analysis and TSS localization in the *CACNG2-DT* promoter. **(A)** Scheme depicting several regulatory elements in *CACNG2-DT*. **(B)** Deletional analysis showing that all constructs have lower transcriptional activity than the complete promoter, except for construct C (ANOVA; *p* < 0.05; *n* = 6).

Subsequently, a deletional analysis was conducted by cloning the full-length promoter or various shorter fragments in the pGL3-Basic vector (Figure 2A), and their transcriptional activity was determined using luciferase assays as described elsewhere (Martínez-Hernández et al., 2013). The results show that all constructs have lower transcriptional activity than the full-length promoter sequence. This was statistically significant in all cases except in the C construct (Figure 2B). Here, it is worth mentioning that the TSS experimentally found in the present report (see below) is located in construct C. Unexpectedly, construct BC exhibited significantly decreased transcriptional activity, despite containing the complete sequence of the C construct. Although the reason for this finding is unknown, it may lie in the presence of transcription repressor elements present in construct B. Indeed, our *in silico* analysis revealed multiple repressive domains, including an array of REST elements and a Ca^2+^ regulatory element-binding factor consensus site (CaRE) (Figure 1; Supplementary Table S6).

**FIGURE 3:**
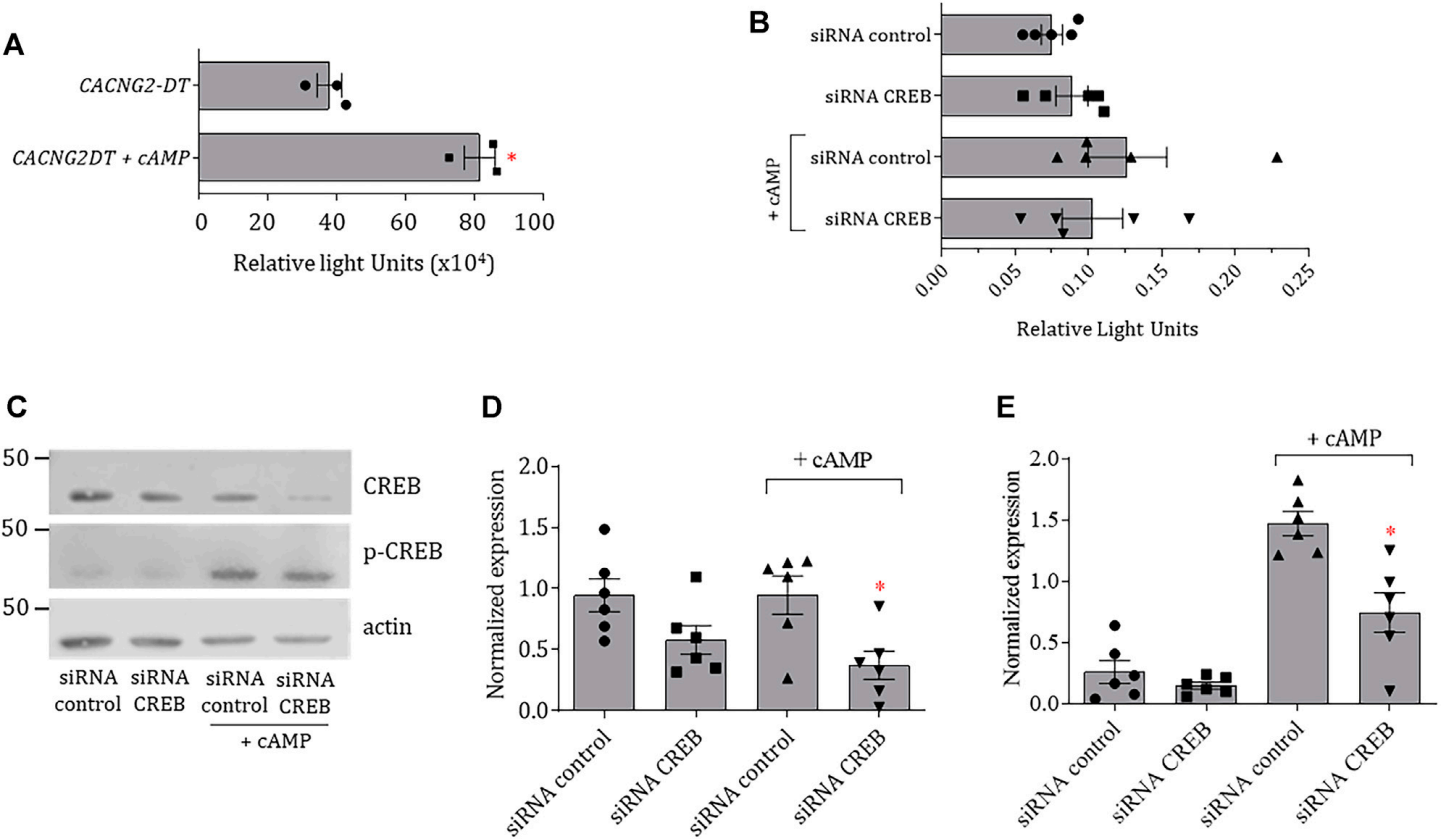
Transcriptional activity of the *CACNG2-DT* promoter and regulation by cAMP and CREB. **(A)** Analysis of the transcriptional activity of the *CACNG2-DT* gene promoter in the absence and presence of cAMP (Student’s t-test; *p* < 0.05; *n* = 3). **(B)** Luciferase assays in protein extracts from N1E-115 cells transfected with siRNAs control and against CREB in the presence or absence of cAMP (Student’s t-test; *p* < 0.05; *n* = 5). Note that the scale of the graphs A and B differs by several orders of magnitude due to the nature of the data normalization (see Statistical analysis). **(C)** Western blot assays of CREB and p-CREB expression in the presence and absence of cAMP. Actin was used as a loading control. **(D,E)** Comparison of the results from the expression of CREB and p-CREB as in C (Student’s t-test; *p* < 0.05; *n* = 6).

In order to further analyze the properties of the *CACNG2-DT* gene promoter, the full-length construct cloned into the pGL3-Basic vector was transfected into N1E-115 cells treated with cAMP, and luciferase assays were performed. The results show that the *CACNG2-DT* promoter exhibited significantly higher transcriptional activity after cAMP treatment (Figure 3A). To begin the study of the molecular mechanism by which cAMP was acting, the effect of the CREB silencing on transcriptional activity was then analyzed. CREB is a transcription factor that binds to DNA sequences (cAMP response elements) and regulates the downstream transcription of several genes. Thus, N1E-115 cells were transfected with the *CACNG2-DT* promoter and small interfering RNAs (siRNAs) designed for CREB silencing (Figure 3B). Cells were then incubated with cAMP to stimulate CREB phosphorylation and assayed for luciferase.

The results of this analysis indicated no significant changes in the transcriptional activity of the promoter in the absence of cAMP. However, there was a significant increase after treatment with the cyclic nucleotide. Interestingly, such an increase is prevented by transfecting the cells with siRNAs against CREB (Figure 3B). To corroborate the correct silencing of CREB, the expression of the transcription factor was analyzed by Western blot both in its inactive and phosphorylated forms (p-CREB). The results showed that transfection with the siRNAs against CREB causes a decrease in the expression of the transcription factor in the presence and the absence of cAMP (Figures 3C, D). On the other hand, the expression of p-CREB was minimal in cells not stimulated with cAMP (Figures 3D, E); however, its levels increased significantly compared to total CREB in the presence of the cyclic nucleotide (Figure 3E). These data suggest that cAMP is stimulating the activity of the *CACNG2-DT* gene promoter acting through the CREB transcription factor.

**FIGURE 4:**
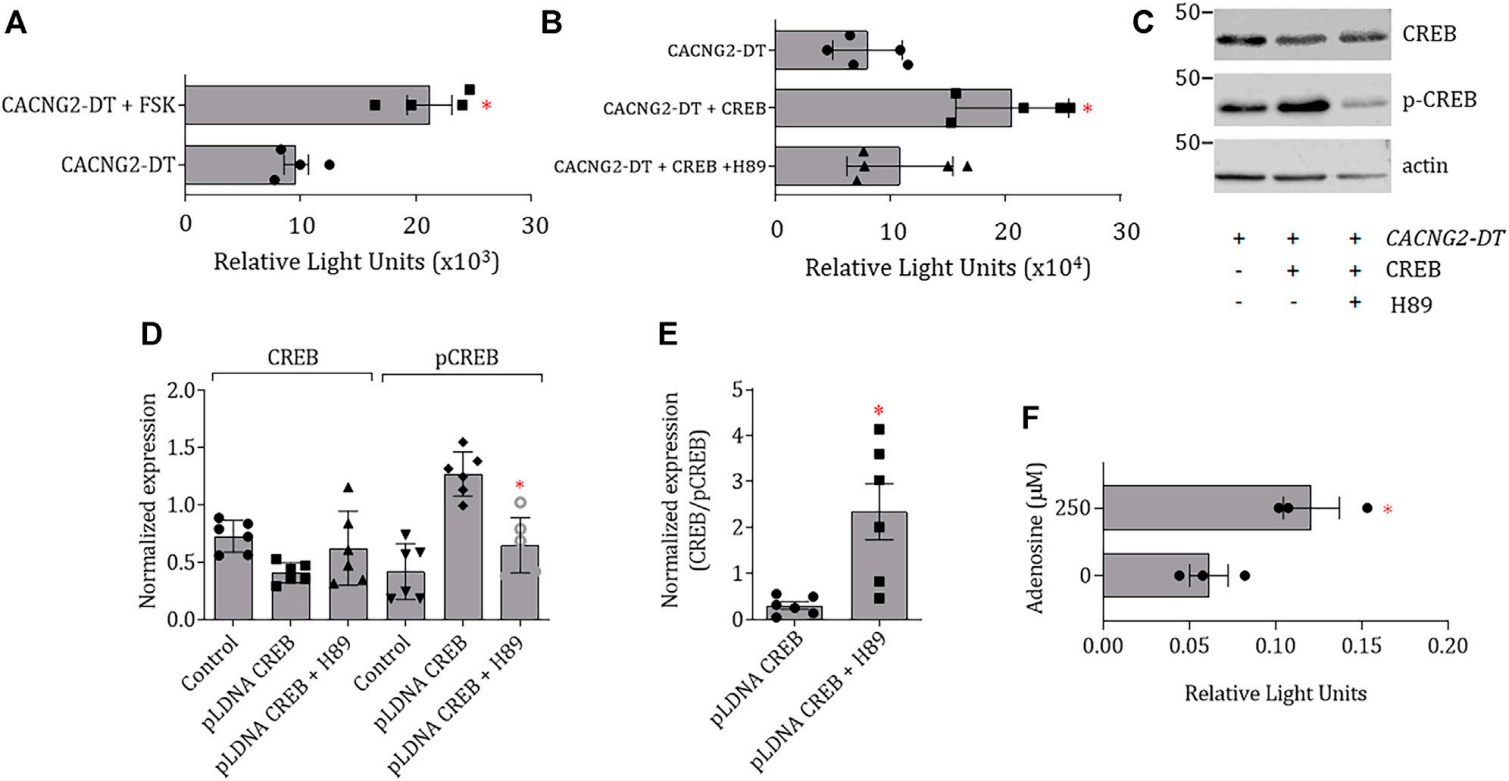
Effect CREB overexpression, AC activation or PKA inhibition on the transcriptional activity of *CACNG2-DT*. **(A,B)** CREB overexpression and FSK incubation cause an increase in the transcriptional activity of the *CACNG2-DT* promoter that is prevented by incubation with H89. **(C)** Western blot images of CREB and p-CREB expression 48 h post-transfection. Actin was used as a loading control (Student’s t-test; *p* < 0.01; *n* = 6). **(D)** Comparison of the expression results of CREB and p-CREB as in **(C)**. **(E)** Comparison of the changes in the CREB/p-CREB ratio after the treatment with H89 as in **(D)**. Treatment with the PKA inhibitor significantly increases CREB/p-CREB indicating the non-active form of CREB is favored in this condition. **(F)** Luciferase assays after activation of a Gs (Adenosine)-coupled receptor, endogenously expressed in N1E-115 cells, showing that the effects of CREB are appreciable *in situ*. (Student’s t-test; *p* < 0.05; *n* = 3). Note that the scale of the graphs **(A)** and **(F)** differs by several orders of magnitude due to the nature of the data normalization (see *Statistical analysis*).

To confirm the involvement of CREB in the regulation of the promoter, the pcDNA3-CREB1 construct was then transfected to assess CREB overexpression in the N1E-115 cells. Transcriptional activity in transfected cells was ∼2.7-fold higher than in untransfected cells. In addition, to gain insights into the signaling pathway used by cAMP to increase transcription, the cells were then incubated in the presence of Forskolin (FSK), an activator of adenylate cyclase, and H89, an inhibitor of PKA. The results showed that the cells treated with FSK (10 μM) have an increased capability to initiate transcription independently of the pcDNA3-CREB1 construct transfection (Figure 4A). In contrast, cells treated with H89 (10 μM) have a decreased capability to initiate transcription even in the presence of pcDNA3-CREB1 (Figure 4B). These findings suggest a mechanism that implies the activation of AC by G protein-coupled receptors and downstream phosphorylation by PKA.

Likewise, in the case of the treatment with H89, the changes in the expression of CREB and p-CREB protein levels were confirmed by Western blot 48 h after transfection (Figure 4C). Figure 4D shows that transfection with pcDNA3-CREB1 significantly increased p-CREB, without affecting the levels of CREB, because most of the CREB had already been phosphorylated before extracting the proteins for the assay. These results suggest that the cAMP-PKA-CREB signaling pathway may be responsible for increasing the transcriptional activity of the *CACNG2-DT* promoter.

Next, given that the cells were transfected with the CREB construct, it proved interesting to observe changes in the CREB/p-CREB ratio and compare this ratio between the control and H89-treated groups. The results of this analysis are shown in Figure 4E, where it can be seen that in the presence of the PKA inhibitor, the CREB/p-CREB ratio is significantly higher, indicating that this condition favors the presence of the non-active form of CREB.

Although incubation with cAMP or forskolin is a reliable method for PKA activation, the question was raised regarding whether these responses could be physiological and reproduced *in situ*. Therefore, the activity of adenylate cyclase was next stimulated by activating a Gs-coupled receptor (adenosine) endogenously expressed in N1E-115 cells (Schrier et al., 2001). The results of this series of experiments are shown in Figure 4F. In this case, N1E-115 cells were transfected with the promoter of the *CACNG2-DT* gene cloned in the pGL3-Basic vector and incubated in the presence of 250 μM adenosine. The results show a significant increase in the transcriptional activity of the promoter after adenosine treatment (Figure 4F).

**FIGURE 5:**
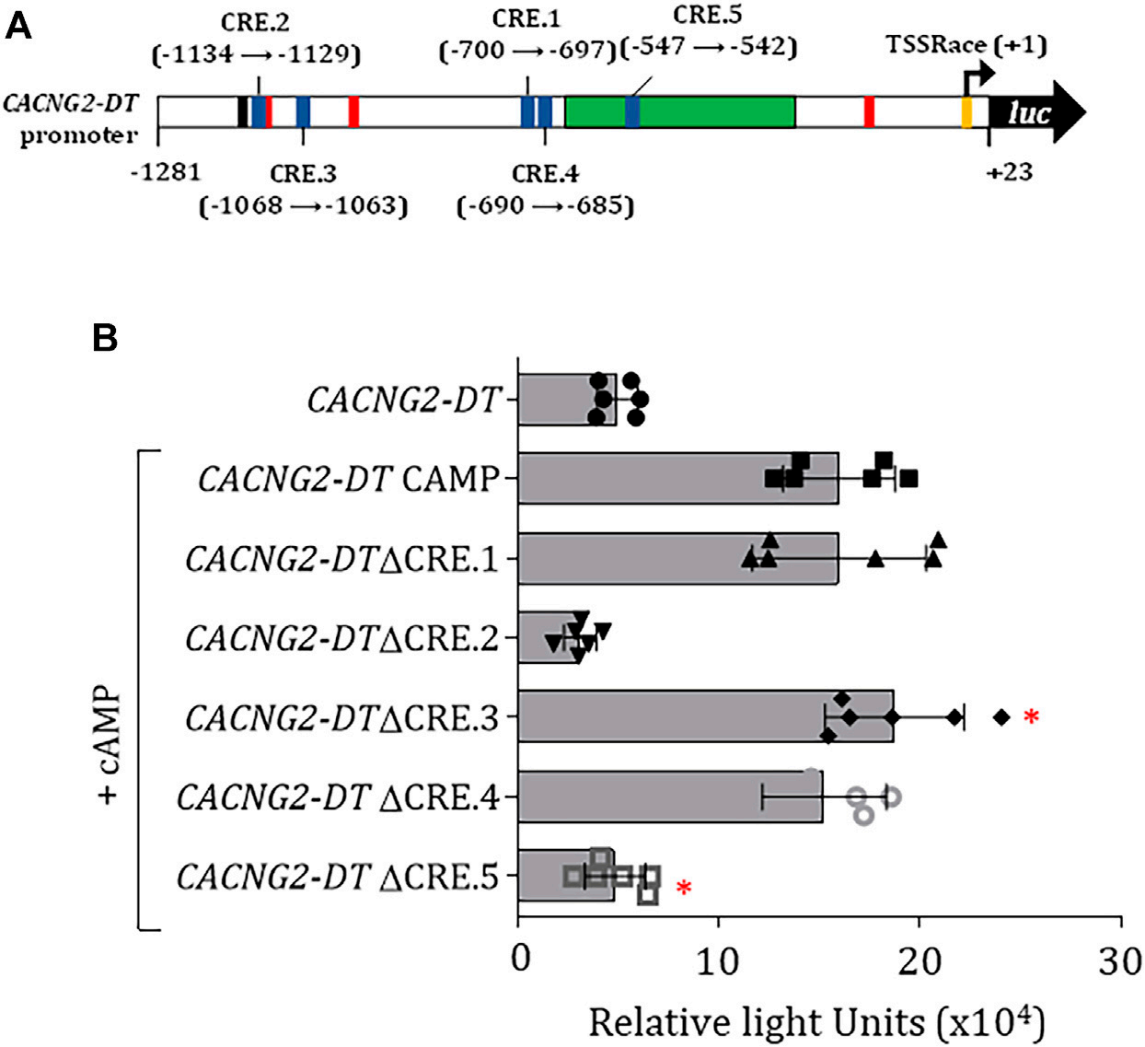
Evaluation of CREB binding sites in the *CACNG2-DT* gene promoter and regulation of Ca_V_γ_2_/Stg expression. **(A)** Scheme of the promoter indicating the location of the CREB binding sites (CRE). **(B)** Luciferase assay under CREB overexpression conditions (+CREB) showing the transcriptional activity of constructs with the elimination of the CREB binding sites (Student’s t-test; *p* < 0.001; *n* = 6).

Next, to identify the CREB-binding sites in the promoter sequence, an *in silico* analysis was performed. This analysis allowed us to identify five sites in the promoter (Figure 5A) which were then removed individually by site-directed mutagenesis. Next, the mutant constructs were transfected into N1E-115 cells and analyzed by luciferase assays. This analysis showed that the constructs with the elimination of the CRE.2 and CRE.5 sites had significantly lower transcriptional activity (Figure 5B).

**FIGURE 6:**
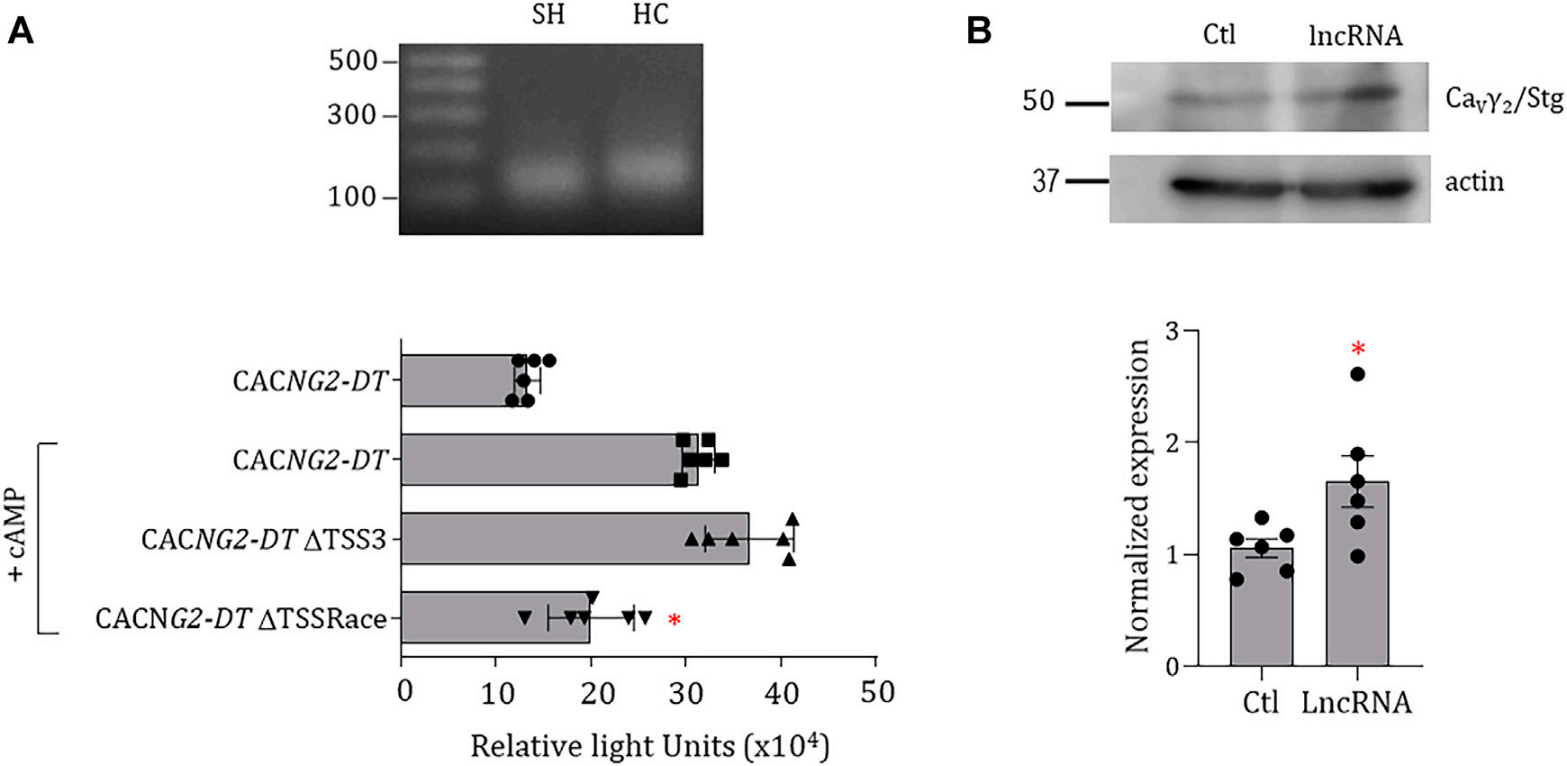
Experimental identification of TSS in the *CACNG2-DT* promoter and effect of the lncRNA on the functional expression of Ca_V_γ_2_/Stg. **(A)** PCR products obtained in the RACE assay. SH: SH-SY5Y cell line; HC: human cerebellum (upper panel). Luciferase assay of the promoter constructs with or without the possible theoretical and experimental TSSs. The assays were conducted under conditions of CREB overexpression (+CREB). The asterisks denote statistically significant differences (ANOVA; *p* < 0.05; *n* = 6). **(B)** Western blot assays of Ca_V_γ_2_/Stg expression in the control condition and in cells expressing *CACNG2-DT* (lncRNA). The lower panel compares the expression level of Ca_V_γ_2_/Stg in the absence and presence of *CACNG2-DT*. The asterisk denotes statistically significant differences (Student’s t-test; *p* < 0.05; *n* = 6).

Likewise, as an essential part of the initial characterization of the promoter, we sought to determine the TSS experimentally. Although the *in silico* analysis identified three potential TSS in the promoter, we proceeded to determine the location of the functional TSS using RNA isolated from SH-SY5Y cells and the human cerebellum. In these experiments, amplicons of ∼150 bp were obtained (Figure 6A, upper panel), and the sequencing revealed that they were the same product. When the sequence of this amplicon was aligned with the theoretical sequence of the promoter a TSS at position +1 (TSSRace) was identified.

As mentioned earlier, the deletional analysis showed that all evaluated constructs showed low transcriptional activity when compared with the full-length promoter except for construct C, where a predicted TSS (TSS3) is located at position -150 according to the *in silico* analysis. For this reason, we then proceeded to compare its functionality with that of TSSRace using luciferase assays by eliminating both sites independently using mutagenesis. This analysis showed that the deletion of the TSS3 does not affect transcription. In contrast, removing the TSSRace decreased the promoter’s ability to initiate transcription (Figure 6A, lower panel), suggesting that transcription of the *CACNG2-DT* gene begins at position +1.

On the other hand, one of the characteristics of bidirectional promoters is that one of the products of the genes regulates the expression of the gene encoded in the opposite direction. To explore this possibility, SH-SY5Y cells were transfected with a vector containing the *CACNG2-DT* exonic sequence, and proteins were extracted for Western blot analysis (Figure 6B, upper panel). The results show that overexpression of *CACNG2-DT* causes a 1.7-fold increase in Ca_V_γ_2_/Stg (Figure 6B, lower panel), suggesting that the lncRNA may upregulate the functional expression of the protein.

## Discussion

**FIGURE 7:**
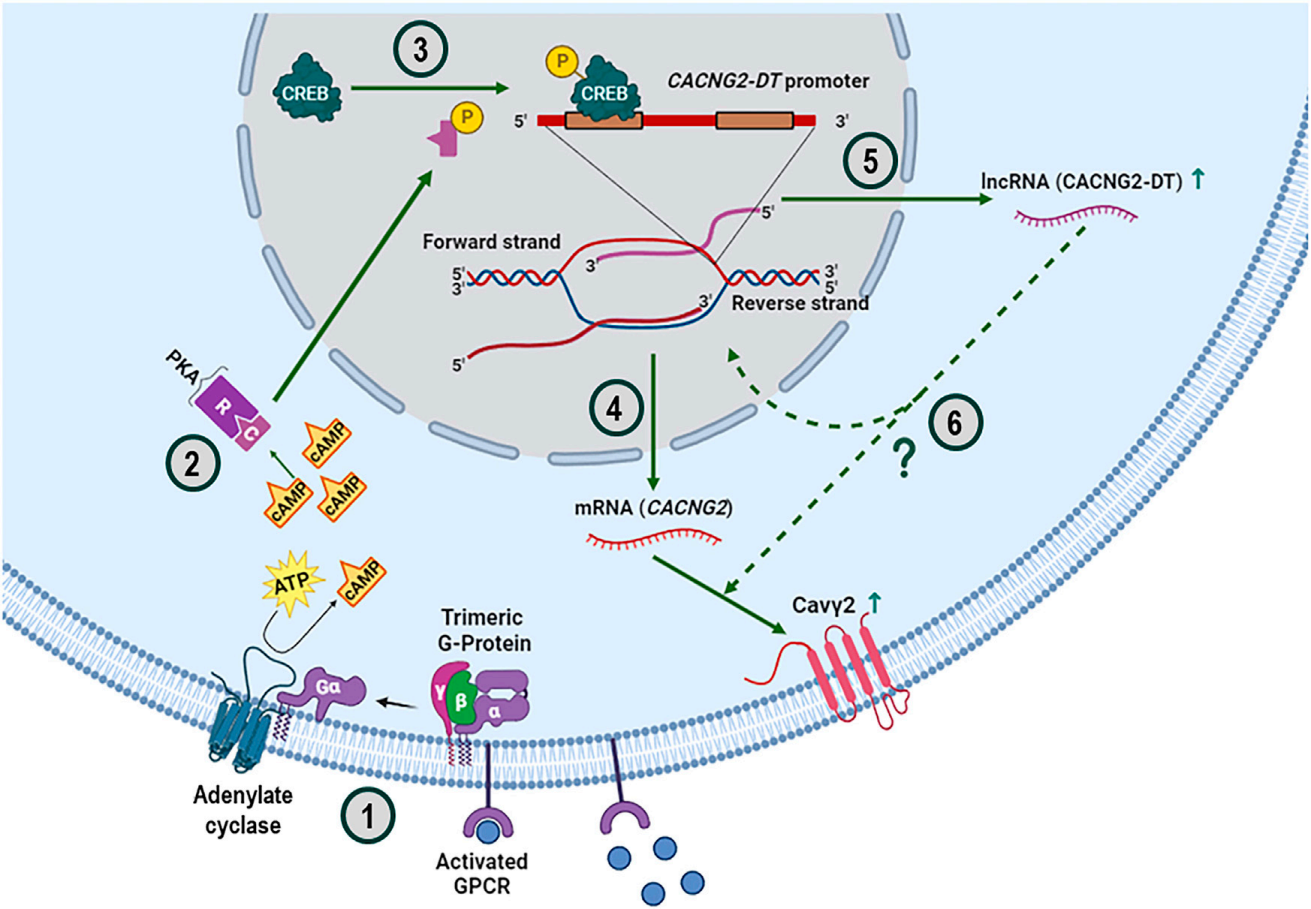
Overview of the cAMP-PKA-CREB signaling pathway and its possible effect on regulating the Ca_V_γ_2_/Stg subunit expression. The upstream activation of trimeric G protein-coupled receptors (GPCRs) results in the dissociation of the Gα subunit, which binds and activates AC to produce the cAMP necessary for signaling through the PKA pathway (1). PKA is a tetrameric holoenzyme comprising two regulatory (R) and two catalytic (C) subunits. When cAMP binds to the R subunits, the enzyme dissociates, allowing the C subunits to phosphorylate the substrates (2). In this case, the C subunits in the nucleus phosphorylate the transcription factor CREB at serine 133, switching it to its active form (3). It is known that phosphorylated CREB binds coactivators to facilitate its interaction with CRE sites and the subsequent transcription of target genes, such as *CACNG2*, which gives rise to the mRNA encoding the Ca_V_γ_2_/Stg subunit (4) and, in the opposite direction of the genome, to the lncRNA called *CACNG2-DT* (5). The overexpression of this lncRNA, by an as yet unknown molecular mechanism, increases the functional expression of Ca_V_γ_2_/Stg (6).

The Ca_V_γ_2_/Stg protein is essential in neuronal signaling due to its association with Ca_V_ channels and AMPA glutamatergic receptors. Consequently, it is unsurprising that alterations in its expression are associated with devastating neurological disorders such as absence epilepsy. However, despite its apparent functional relevance, the regulation of Ca_V_γ_2_/Stg expression has not been investigated in detail. This report shows evidence that 1) the human *CACNG2* promoter, like in the rat, is bidirectional and regulates the transcription of a lncRNA (*CACNG2-DT*) in the antisense direction; 2) the transcriptional activity of the gene promoter encoding *CACNG2-DT* may be greater than that of *CACNG2*; 3) the TSS is located at position +1 of the gene promoter encoding *CACNG2-DT*; 4) the cAMP-PKA/p-CREB signaling pathway is involved in the transcriptional regulation of *CACNG2-DT*; 5) the active form of CREB (p-CREB) could be regulating the activity of the gene promoter encoding *CACNG2-DT* through its binding to at least two CREB sites; and 6) *CACNG2-DT* may regulate the Ca_V_γ_2_/Stg protein expression levels (Figure 7).

Our *in silico* analysis provided evidence for the role of the REST transcription factor (Garcia-Manteiga et al., 2019) in regulating *CACNG2-DT* expression, corroborating previous data showing the direct regulation of rat *Cacng2* by REST. Our analysis showed a distal repressor region containing multiple REST elements at positions -665 to -564 and a CaRE site at position -495 to -485. The decrease in transcriptional activity observed in construct BC in the deletional analysis may be caused by the repressor elements present in construct B (Figure 1) and could be evidence of the regulatory ability of these elements on the promoter activity *in vivo*. Indeed, previous studies have shown that selective deletion of the REST and CaRE domains increases promoter-driven transcription. For instance, to directly evaluate the role of REST repressor sequences in the promoter, a dominant-negative (DNR) REST construct has been used whose transfection into cell lines results in a marked increase in transcriptional activity, indicating a derepression of the promoter (Park et al., 2007; Corney et al., 2019).

On the other hand, in the rat genome, it has been reported that the *Cacng2* gene encoding Ca_V_γ_2_/Stg, in the opposite direction, gives rise to two lncRNAs of 405 and 578 bp (Corney et al., 2019). However, our study experimentally identified a single TSS at position +1 of the gene promoter of interest that control the expression of a 556 bp lncRNA. It should be mentioned, however, that the *in silico* analysis using the Ensembl database suggests the presence of an additional TSS at position -57 that would control the expression of an 855 bp isoform (ENST00000430281.3). Nevertheless, we could not confirm the presence of such a site experimentally. The reason for this discrepancy is also unknown; however, it may lie in the fact that this isoform might not be expressed in the cell line or the tissue used in our study. It is worth noting, however, that the TSS and the isoform mentioned above have only been found *in silico*. To our knowledge, no experimental evidence has been yet provided.

Likewise, transcriptional regulation through the binding of transcription factors to the cAMP response element (CRE) downstream of cAMP signaling plays important roles in many cellular processes. Transcription factors that bind to CRE, such as CRE-binding protein (CREB), are activated by cAMP-dependent PKA-mediated phosphorylation and stimulate gene expression by recruiting coactivators. Our study provides experimental evidence for the role of CREB in regulating *CACNG2-DT* expression. The presence of functional CRE sites implies the activation of trimeric G protein-coupled membrane receptors and the subsequent activation of protein kinases that phosphorylate multiple targets.

It is worth mentioning here that CREB has many functions in different organs and tissues, including the brain, where its activity is related to learning and memory (Wu et al., 2007) where NMDA and AMPA receptors play a decisive role. Therefore, it would be interesting to investigate whether the expression of *CACNG2-DT* could play a role in this process. On the other hand, investigating a potential association of these CREB elements with seizure phenotypes and pain-related plasticity in which Ca_V_γ_2_/Stg has been implicated (Letts et al., 1998; Nissenbaum, 2012; Henley and Wilkinson, 2016) is also an exciting topic for future studies.

Another aspect to highlight has to do with the bidirectional nature of the promoter. It is well known that many promoters with this characteristic may give rise to a non-coding RNA and a protein-coding messenger (Adachi and Lieber, 2002; Jiménez-Badillo et al., 2017). Interestingly, there is evidence that non-coding RNAs could modify the expression of the protein encoded in the opposite direction (Wei et al., 2011). Our results suggest that the expression of *CACNG2-DT* increases the Ca_V_γ_2_/Stg protein levels, reinforcing the idea that the promoter of the *CACNG2* gene is bidirectional and that one of its gene products is a lncRNA. This finding further provides experimental evidence for the regulated co-expression of the lncRNA-protein pair reported for other similarly organized genes (Guttman and Rinn, 2012; Hombach and Kretz, 2016; Bridges et al., 2021; Felix et al., 2022).

Although the molecular mechanism by which *CACNG2-DT* increases the Ca_V_γ_2_/Stg protein functional expression remains unclear, several possibilities exist to explain it. On the one hand, lncRNA could act in the nucleus regulating the transcriptional machinery. In addition, the *CACNG2-DT* could be involved in epigenetic regulation processes of gene transcription activation by modulating chromatin accessibility (Guttman and Rinn, 2012; Hombach and Kretz, 2016; Bridges et al., 2021). On the other hand, once in the cytoplasm, *CACNG2-DT* could participate in diverse molecular processes, such as the modulation of signal translation pathways that favor the functional expression of the protein, the regulation of the mRNA’s stability and/or the regulation of the accessibility of miRNAs that control their expression (Guttman and Rinn, 2012; Hombach and Kretz, 2016; Bridges et al., 2021). All these possibilities are attractive for future studies. Interestingly, it is known that miRNAs may directly interact with complementary sites in the 3′ untranslated region of target mRNAs and repress their expression (Bushati and Cohen, 2007). In this context, it is reasonable to speculate that *CACNG2-DT* could interact with a miRNA that downregulates the functional expression of Ca_V_γ_2_/Stg and, therefore, promotes an increase in protein levels. However, experimental evidence is still required to support this notion.

## Data Availability

The raw data supporting the conclusion of this article will be made available by the authors, without undue reservation.
