# Supplementary material for: Molecular cloning of the gene promoter encoding the human CaVγ2/Stargazin divergent transcript (CACNG2-DT): characterization and regulation by the cAMP-PKA/CREB signaling pathway
Source: Front Physiol. 2023 Nov 16;14:1286808. doi: 10.3389/fphys.2023.1286808 (PMC10687476; doi:10.3389/fphys.2023.1286808)
Supplement: Supplementary file 1 [file Table5.pdf]

SUPPL. TABLE 5. Data normalization method used in the transcriptional activity evaluation assays.

Figure	Transfection	Normalization
2B	pGL3-Basic	β-Galactosidase
3A	pGL3-Basic	β-Galactosidase
3B	pGL3-Basic	*Renilla* luciferase
4A	pGL3-Basic	β-Galactosidase
4F	pGL3-Basic	*Renilla* luciferase
5B	pGL3-Basic	β-Galactosidase
6A	pGL3-Basic	β-Galactosidase
