# Supplementary material for: Molecular cloning of the gene promoter encoding the human CaVγ2/Stargazin divergent transcript (CACNG2-DT): characterization and regulation by the cAMP-PKA/CREB signaling pathway
Source: Front Physiol. 2023 Nov 16;14:1286808. doi: 10.3389/fphys.2023.1286808 (PMC10687476; doi:10.3389/fphys.2023.1286808)
Supplement: Supplementary file 2 [file Table4.pdf]

SUPPL. TABLE 4. Oligonucleotides used in the 5'RACE technique.

Oligonucleotide	Sequence (5' → 3')
GSP1 AL0	GTCCAGGTTACATGTT
GSP2 AL0	TTCCAGGCAGAGAAAC
NGSP AL0	GTTGAAGTTTTGTGCC
