# Supplementary material for: Molecular cloning of the gene promoter encoding the human CaVγ2/Stargazin divergent transcript (CACNG2-DT): characterization and regulation by the cAMP-PKA/CREB signaling pathway
Source: Front Physiol. 2023 Nov 16;14:1286808. doi: 10.3389/fphys.2023.1286808 (PMC10687476; doi:10.3389/fphys.2023.1286808)
Supplement: Supplementary file 3 [file Table1.pdf]

SUPPL. TABLE 1. Oligonucleotides used for the deletional analysis of the CACNG2-DT gene promoter.

Construct	Base pair (Start ⟶ End)	Oligo	Sequence (5' → 3')
CACNG2-DT promoter	1304 bp (-1281 ⟶ +23)	A Fw<br>C Rev	TTTTTCTCGAGCACCCAACCGACTTCTGGTT<br>TTTTTAAGCTTCTCTGGGCACGTAGAGATGG
A	414 bp (-1281 ⟶ -868)	A Fw<br>A Rev	TTTTTCTCGAGCACCCAACCGACTTCTGGTT<br>TTTTTAAGCTTATGGTGTTGAGAATTCGGCT
B	450 bp (-867 ⟶ -418)	B Fw<br>B Rev	TTTTTCTCGAGTTCTCATGGTCGGGACCTAGACA<br>TTTTTAAGCTTGCCCACCCTGCAGCAAGACG
C	440 bp (-417 ⟶ +23)	C Fw<br>C Rev	TTTTTCTCGAGCGCGCCTGCCCCCACTC<br>TTTTTAAGCTTCTCTGGGCACGTAGAGATGG
AB	864 bp (-1281 ⟶ -418)	A Fw<br>B Rev	TTTTTCTCGAGCACCCAACCGACTTCTGGTT<br>TTTTTAAGCTTGCCCACCCTGCAGCAAGACG
BC	890 bp (-867 ⟶ +23)	B Fw<br>C Rev	TTTTTCTCGAGTTCTCATGGTCGGGACCTAGACA<br>TTTTTAAGCTTCTCTGGGCACGTAGAGATGG

The poly T-tail and the added cleavage sites for Xho and HindIII endonucleases (for forward and reverse oligonucleotides, respectively) are underlined in the sequence.
