# Supplementary material for: Molecular cloning of the gene promoter encoding the human CaVγ2/Stargazin divergent transcript (CACNG2-DT): characterization and regulation by the cAMP-PKA/CREB signaling pathway
Source: Front Physiol. 2023 Nov 16;14:1286808. doi: 10.3389/fphys.2023.1286808 (PMC10687476; doi:10.3389/fphys.2023.1286808)
Supplement: Supplementary file 4 [file Table2.pdf]

SUPPL. TABLE 2. Oligonucleotides used for cloning of CREB binding sites (CRE).

Site	Oligo	Sequence (5' → 3')
CRE.1	Fw	GACCAGACTTCCCAGTATTCTGCCCTTGAGCT
	Rev	AGCTCAAGGGCAGAATACTGGGAAGTCTGGTC
CRE.2	Fw	GAATATGGAGAGTTATAAAAAAAGGAGAAAGCTCACGGAAAAGAGTG
	Rev	CACTCTTTTCCGTGAGCTTTCTCCTTTTTTTATAACTCTCCATATTC
CRE.3	Fw	GGGAAGAGGCTTGCCTTTTAGAAACTGTTCCAGT
	Rev	ACTGGAACAGTTTCTAAAAGGCAAGCCTCTTCCC
CRE.4	Fw	CCCAGTATTCTGTAAGCCCTTAGCTGCACAGGTG
	Rev	CACCTGTGCAGCTAAGGGCTTACAGAATACTGGG
CRE.5	Fw	GGCGGCGGTTATTGTTGTTGGGGGTAGTG
	Rev	CACTACCCCCAACAACAATAACCGCCGCC
