# Supplementary material for: Molecular cloning of the gene promoter encoding the human CaVγ2/Stargazin divergent transcript (CACNG2-DT): characterization and regulation by the cAMP-PKA/CREB signaling pathway
Source: Front Physiol. 2023 Nov 16;14:1286808. doi: 10.3389/fphys.2023.1286808 (PMC10687476; doi:10.3389/fphys.2023.1286808)
Supplement: Supplementary file 5 [file Table6.pdf]

SUPPL. TABLE 6. Sequence and relative position of the REST and CaRE elements/regions.

Site / Element	Base pair (Start ⟶ End positions)	Sequence (5' → 3')
REST (1)	22 bp (-665 ⟶ -644)	TCTCGCTTTCCATGGTTTTGCC
REST (2)	25 bp (-646 ⟶ -622)	GCCCGGGCAGCGGCGGCGGCGGCGG
REST (3)	25 bp (-643 ⟶ -619)	CGGGCAGCGGCGGCGGCGGCGGCGG
REST (4)	25 bp (-637 ⟶ -613)	GCGGCGGCGGCGGCGGCGGCGGCGG
REST (5)	25 bp (-634 ⟶ -610)	GCGGCGGCGGCGGCGGCGGCGGCAG
REST (6)	21 bp (-584 ⟶ -564)	GGCAGCAGGACGAGCAGCGGC
CaRE	11 bp (-495 ⟶ -485)	GAATCGAGGCG
