# Supplementary material for: Molecular cloning of the gene promoter encoding the human CaVγ2/Stargazin divergent transcript (CACNG2-DT): characterization and regulation by the cAMP-PKA/CREB signaling pathway
Source: Front Physiol. 2023 Nov 16;14:1286808. doi: 10.3389/fphys.2023.1286808 (PMC10687476; doi:10.3389/fphys.2023.1286808)
Supplement: Supplementary file 6 [file Table3.pdf]

SUPPL. TABLE 3. siRNAs used for CREB knockdown.

Oligo	Site	Sequence
siRNA CREB 1a	CREB binding site	5’-CAGCCAUCAGUUAUUCAGU-3’<br>5’-ACUGAAUAACUGAUGGCUG-3’
siRNA CREB 1b	CREB binding site	5’-GAGCAAUACAGCUGGCUAA-3’<br>5’-UUAGCCAGCUGUAUUGCUC-3’
